# Hospital Meetings, &C.

**Published:** 1898-04-30

**Authors:** 


### HOSPITAL MEETINGS, &C.

### ROYAL HOSPITAL FOR DISEASES OF THE CHEST.

Mr. S. Hope Morley, the treasurer, presided over the festival dinner of the Royal Hospital for Diseases of the Chest, which was held at the Whitehall Rooms on Thursday, 21st inst. The object of the dinner was to raise £7,000 in order to clear off the loan advanced by the bankers (£800), to pay accounts owing to tradespeople, and to enable the Council to carry on the work of the hospital. As a result of the festival £4,400 was raised, the highest amount, with the exception of the sum contributed when the Prince of Wales presided in 1893, ever collected at a dinner in aid of the hospital. The Chairman's list, including a contribution of £500 from himself, totalled about £3,000. The Chairman, in proposing the toast of the evening, "Success to the Hospital," traced the history of the hospital from its foundation in 1814 by the Duke of Kent, the father of the Queen, as the "Infirmary for Diseases of the Lungs," down to the present day, and showed the difficulties which had been surmounted by its supporters. The past year, he said, had been a very trying one for many an institution like theirs, and their resources had been diminished, when compared with 1896, by no less than £3,900. He could not, however, believe but that their friends would rally round them again when the Jubilee year and its attendant expenses had been forgotten, and that if some few of the older friends had been lost to the institution, there were younger ones ready to take their place. Mr. T. Andros de la Rue, the chairman of the council, proposed the health of the new president, Lord Rothschild. Other toasts followed.

### ROYAL HOSPITAL FOR CHILDREN AND WOMEN.

Mr. H. M. Stanley, who is M.P. for the division in which the Royal Hospital for Children and Women is situated, was one of the guests at the festival dinner which was held at the Hotel Cecil on Wednesday, 20th inst., under the presidency of Sir Edwin Durning-Lawrence, Bart., M.P. In replying to the toast of "The Visitors," he drew a graphic picture of the privations which he had undergone in Central Africa, and said that his experience on one occasion, when as he and his companions were on the verge of starvation food had appeared in an almost miraculous fashion, made him realise to the full what were the sufferings of the poor in the South of London, and with what joy they must welcome the relief afforded them by a hospital such as the one whose cause they were gathered together to assist that evening. The Chairman, in proposing the loyal toasts, said that the hospital had been tenants of the Prince of Wales, and were the only tenants on his estates who had been granted the right of pre-emption. Later in the evening, in proposing "The Royal Hospital for Children and Women," he said that people always spoke of the poverty of the East End of London, but they did not seem to realise that the poorest part of the metropolis was on the south side of the Thames. In Lambeth, for instance, there was more misery, he believed, than could be found in the East End. It was in such a district that the hospital was doing its work, and being almost entirely dependent on voluntary contributions, it must be supported day by day and hour by hour by the gifts of the public. In responding, the Treasurer (Mr. John F. Eastwood) said that if the hospital could get a further £2,000 a year in annual subscriptions, it would be freed from all difficulty. Such a sum could easily be found were only a small part of the gentlemen who arrived at Waterloo every morning, and then hurried past the hospital to their work in the City, to contribute to its funds. Contributions amounting to about £670 were announced as the result of the dinner.

### ST. MARK'S HOSPITAL.

As the Duke of Connaught, who was to have presided, was unable to be present, the Lord Mayor took the chair at the festival dinner to celebrate the sixty-third anniversary of St. Mark's Hospital, which was held at the Whitehall Rooms on Monday, the 25th inst. Amongst those present were Viscount Dungarvan, M.P., Mr. R. Biddulph Martin, M.P., (treasurer), Sheriffs Green and Dewar, Colonel Mitford, and Archdeacon Sinclair. In proposing the toast of "Prosperity to St. Mark's Hospital," the Lord Mayor said that it was a great misfortune to the hospital that the Duke of Connaught was not able to be present, but his Royal Highness had shown his personal interest by sending a contribution of £25. Founded in 1835 at Aldersgate Street, the hospital was moved to Charterhouse Square in 1837, and to the present site in the City Road in 1851, on each occasion greatly increasing the number of beds. Pressure on the beds, however, continued, and from the seventies until 1894 the committee were trying to raise funds to rebuild the institution. In the latter year the work was commenced, and in 1897 the new building was opened. The beds now numbered 42. The cost of this new building was met out of the invested funds, and the income in consequence had been reduced. That dinner, he said, differed from the hardy annuals which bloomed about this time every year, because they had not had a festival for 22 years, and in consequence of this, and because the hospital was a valuable one, he hoped the charity would receive their support. Mr. R. Biddulph Martin, in responding, said that the hospital received patients from all parts of the kingdom, and that the new buildings were excellent. Other toasts followed, in the course of which the Secretary announced that the result of the dinner had been the collection of £2,094.

### WORKING MEN AND HOSPITALS.

The third of the series of meetings arranged by a joint committee of the Federation of Working Men's Clubs and the Club and Institute Union was held at the offices of the London Chamber of Commerce on Wednesday, the 20th inst. Mr. Eyre presided, and the subject considered was the relation of provident dispensaries and similar institutions to the hospitals.

Mr. Warren, secretary of the Metropolitan Provident Medical Association, who was the first speaker, observed that the provident dispensaries occupied a more prominent position than ever in relation to hospital reform. He then proceeded to state that in 1896 the number of separate out patients at 26 general hospitals was 943,248, and at the special hospitals 404,651. These figures struck him as being somewhat appalling, and if they were correct something should be done to remedy this state of affairs. All authorities were agreed on the point. He had said all authorities, but perhaps this was not quite so, for he remembered a speech by Sir Henry Burdett, and he was not quite clear from that gentleman's remarks that reform was necessary. He (Sir Henry) seemed to think these huge figures justified rather than condemned the out-patient departments. Proceeding to describe the aims of the Metropolitan Provident Medical Association, Mr. Warren stated that it was established in 1881 to co-operate with the governing bodies of the hospitals, and to enable the working classes to provide for their own medical wants. The free relief provided by the hospitals tended to strangle the provident dispensary. But the question was, Could the hospitals and the provident dispensaries co-operate? The effect of proper co-operation would certainly be to reduce the number of out-patients; indeed, he believed the provident dispensary offered the only safe solution of the out-patient problem. It seemed to be thought that if this reduction took place there would not be a sufficient number of clinical cases, but if real co-operation existed there would be no deficiency of material. The weak point in the provident dispensary system was that it was a form of insurance, and consequently when a person suffering from actual illness desired to join, a fee practically prohibitive to an ordinary working man was demanded. If the co-operation he had spoken of was to be real, the Provident dispensaries must take steps to alter this. Mr. Warren went on to state that the Royal Free Hospital did co-operate with his association, and that the results were very satisfactory. The almoner furnished the association with reports, and in cases where the people concerned were fit subjects for provident dispensaries they were called upon by the association's collectors or visitors. The association also co-operated with the Hospital Saturday Fund, its medical officers undertaking to examine applicants for letters for chest hospitals. In 2,069 cases where persons applying for letters had received instead forms to be examined, 622 were judged to be eligible, 64 were provided with letters for other hospitals, and 367 were considered suitable for provident dispensaries. In 957 instances the forms never came back. Mr. Warren concluded by emphasising the point that co-operation with the hospitals would not be effected unless uniformity and elasticity were introduced into the management of the dispensaries generally. A working man, for instance, removing from one part of London to another should at once be able to derive medical benefit from a dispensary in his new locality.

In reply to a question as to whether those who joined provident dispensaries were thriftless people with large families, who thought this the cheapest way of obtaining medical advice, Mr. Warren replied in the negative. The great majority joined, he said, because they were provident and thrifty, and in the most successful dispensaries a large proportion of the members were members of friendly societies. His association had a scheme for transferring members from one district to another.

Colonel Montefiore, who was next called upon to speak, asserted that the whole matter lay in the hands of the working men, and if they were determined to act on provident lines, they would soon get the doctor to act with them. The hospital should be a charity pure and simple. To-day the hospitals had the best medical men, and among these were young men who were anxious—and rightly so—to climb the ladder. To do this they wanted to examine as many patients as they could, and it was not to be expected that the lay element would move when the doctors were desirous of having as large a number of cases as possible. What the working men wanted was the good old family doctor, and they could get him by combining. To the doctor at the hospital the patient was only, say, "Number 22," but the provident doctor would be the friend of his patients. Mr. Sydney Holland had spoken of payments made at the hospitals and seemed to think they were provident payments. They were nothing of the kind. At the hospitals people of all descriptions were mixed up together, and they were "hail fellow, well met," so long as they were cases.

Mr. Hamilton Hoare, in a brief speech, said that if the working men were to combine as they did in their trade unions and their friendly societies, they could establish a system outside the hospitals, which they would manage themselves.

Mr. B. T. Hall, of the Club and Institute Union, observed that there appeared to be some confusion somewhere, for while it was now said the hospitals wanted patients, Mr. Sydney Holland's grievance was that they had too many. He believed that a strong organisation, able to speak authoritatively to the hospitals, would be the right thing, though he appreciated the difficulties that stood in the way of its formation.

The proceedings shortly afterwards terminated, it having been arranged that at the next meeting the whole question should be summed up and the establishment of a Central Hospital Board discussed. It was further suggested that Sir William Broadbent, Sir Henry Burdett, and Mr. Loch, of the Charity Organisation Society, might be invited to attend, but the final arrangements were left in the hands of the secretaries.

